# Whole exome capture in solution with 3 Gbp of data

**DOI:** 10.1186/gb-2010-11-6-r62

**Published:** 2010-06-17

**Authors:** Matthew N Bainbridge, Min Wang, Daniel L Burgess, Christie Kovar, Matthew J Rodesch, Mark D'Ascenzo, Jacob Kitzman, Yuan-Qing Wu, Irene Newsham, Todd A Richmond, Jeffrey A Jeddeloh, Donna Muzny, Thomas J Albert, Richard A Gibbs

**Affiliations:** 1 Human Genome Sequencing Center, Baylor College of Medicine, One Baylor Plaza, Houston, Texas 77030, USA; 2 Department of Structural and Computational Biology and Molecular Biophysics, Baylor College of Medicine, One Baylor Plaza, Houston, Texas 77030, USA; 3 Roche NimbleGen, Inc., 504 S. Rosa Road Madison, WI 53719, USA

## Abstract

We have developed a solution-based method for targeted DNA capture-sequencing that is directed to the complete human exome. Using this approach allows the discovery of greater than 95% of all expected heterozygous singe base variants, requires as little as 3 Gbp of raw sequence data and constitutes an effective tool for identifying rare coding alleles in large scale genomic studies.

## Background

DNA sequence capture is an effective technique for enrichment of significant subfractions of the genome for targeted analysis. Sequence capture is generally conducted with either a solid-phase substrate, usually a glass microarray, or alternatively, in solution [1-4]. Solution-capture performance, however, has not been thoroughly compared to microarray with respect to uniformity of representation of the targeted DNA bases or evenness of DNA sequencing coverage depth between regions [5,6]. Further, solution-based sequence capture has not been demonstrated to work effectively at the scale of a human exome (approximately 30 Mbp) but typically has been limited to targets <5 Mbp in size. Despite this, solution capture has several advantages when compared to microarrays: the reagent cost is lower; less DNA is required; and, because the capture method can be conducted entirely in small laboratory tubes, it is readily scaled and automated. Before solution-capture sequencing can be widely adopted, however, the reproducibility of the method must first be demonstrated, and targets should show similar levels of coverage from capture to capture. Ideally, solution-capture methods should also be able to be coupled to different sequencing technology platforms, and reliably produce suitable levels of enrichment that routinely enable the discovery of rare genetic variants.

This report is the first demonstration of whole exome capture in solution (Table 1). We demonstrate similar levels of specificity to microarray-based techniques without sacrificing reproducibility or specificity of either the capture or variant discovery while maintaining all the advantages of solution-based techniques over microarray capture.

**Table 1:** A comparison of different capture methodologies

Study	Capture type	Reactors	Capture size	Sequencer type
Ng *et al. *[3]	Array	Multiple array	>30 Mbp	Illumina
Choi *et al. *[11]	Array	Single array	>30 Mbp	Illumina
Gnirke *et al. *[5]	Solution	Single tube	<5 Mbp	Illumina
This study	Solution	Single tube	>30 Mbp	SOLiD/Illumina

To test the reproducibility of our recent innovations of liquid DNA capture, technical replicate capture experiments were performed and subsequently sequenced on the SOLiD [7] platform. Capture followed by Illumina [8] sequencing was also performed with both a fragment (frag) and paired-end (PE) library to test the merits of employing PE data versus single-ended reads. Finally, we used each of these data sets to test the ability to discover single nucleotide variants across the exome.

## Results and discussion

Here we report the performance of newly developed methods for sequence-capture in solution. The procedures were tested with respect to reproducibility of capture, agnosticism to sequencer platform, and the ability to discover genetic variation in human gene coding regions. In total, six captures of the Consensus Coding Sequence (CCDS) exons [9,10] were performed using DNA from one HapMap sample (NA12812). Four separate, replicate capture libraries were prepared for solid sequencing and one additional library was prepared for both the Illumina frag and PE sequencing. In total, 23 Gbp of data were uniquely aligned to the human reference genome (Table S1 in Additional file 1). Variants were called using algorithms tailored to each sequence platform and corresponding data type, and compared to known variants in the HapMap sample (see Materials and methods).

**Figure 1:**
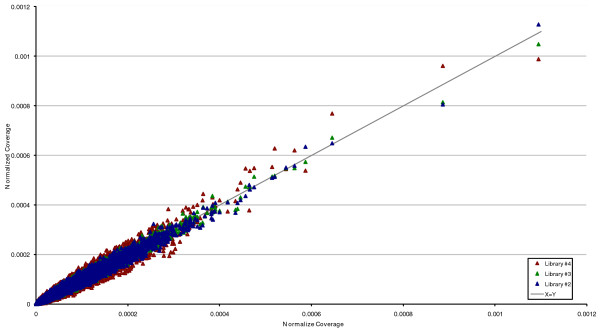
**Normalized coverage of replicate SOLiD libraries 2 to 4 versus normalized coverage of replicate library 1**. Average coverage for each target region in library 1 is plotted against library 2 (blue), library 3 (green) and library 4 (red). Coverage for each target is represented as a proportion of the total sequence generated. The line X = Y indicates approximately equal levels of coverage in both libraries.

To first test the reproducibility of the entire capture-sequencing process, four technical replicate solution captures were performed. Each replicate was sequenced using one-quarter of the same SOLiD DNA sequencer 'slide' to eliminate run-by-run sequencer variability. The standard procedure for categorizing data from this sequencing platform is to identify individual sequence reads that can be mapped at high stringency to a reference sequence. These 'mappable' reads constitute the usually cited yield for each sequence run. Here, the sequences from four technical replicate libraries had an average of 49.6% (standard deviation 1.23) of mappable reads derived from the capture target regions with the remainder mapping elsewhere to the genome. This efficiency of properly targeted sequence reads represents a value similar to Ng *et al. *[3], and higher than Choi *et al. *[11]. The final DNA sequence coverage across each target had >98% correlation between all four libraries (Figure 1). In three of four experiments, >65% of the targeted bases were covered ten or more times, and the observed variation in the coverage levels was primarily accounted for by the total sequence yield of each spot. These results indicate that, for a given amount of sequence data, the average coverage and distribution of coverage is highly predictable and the performance of each individual target region in different experiments is consistent.

One technical artifact of capture-sequencing procedures is the generation of duplicate DNA sequencing reads that represent the repeated sequencing of copies of the same molecule. These duplicates generally arise when there are too few total molecules present at any stage of the technical manipulations - especially immediately prior to any PCR step. Detection of the duplicate reads by computational analysis is not trivial, and generally relies on observation of the alignment positions. Unfortunately, these artifactual duplicates are difficult to distinguish from exactly overlapping reads that naturally occur within deep sequence samples.

The percentage of duplicate reads, defined as reads with the same start point and direction, was approximately 22% for 3 Gbp of aligned data and was also highly consistent between replicates (Table 2, libraries 2 to 4). As expected, when more reads were generated for the SOLiD library 1 the duplicate rate increased to approximately 33%. Simulations of these data showed an expected 'natural' (that is, not PCR-induced) duplication rate of 22 and 14% for 10 Gbp and 3 Gbp of data, respectively. This means that approximately 33% of our suspected duplicate reads are likely not due to PCR-induced duplication, but are those expected from the random distribution of read ends. As noted above, it is not possible to differentiate true duplicate reads from randomly occurring duplicates, and thus all such reads were removed prior to further analysis.

**Table 2:** Alignment statistics for SOLiD frag sequencing libraries

	SOLiD library			
	
	1	2	3	4
Total reads aligned	199,704,874	53,558,797	47,640,266	46,687,848
Total data aligned (Gbp)	9.99	2.68	2.38	2.33
Reads on target (%)	50.1	50.9	49.5	48.0
Duplicate reads (%)	32.8	22.1	22.5	20.9
Mean coverage (X)^a^	43	22	20	19
Median coverage (X)^a^	42	19	16	15
Targets hit (%)	99.3	98.44	98.4	98.1
Bases ≥1× coverage (%)	97.6	94.31	93.5	92.5
Bases ≥10× coverage (%)^a^	89.4	70.8	65.9	64.1
Bases ≥20× coverage (%)^a^	78.9	48.2	42.0	40.5

^a^Calculated after duplicate read removal.

**Figure 2:**
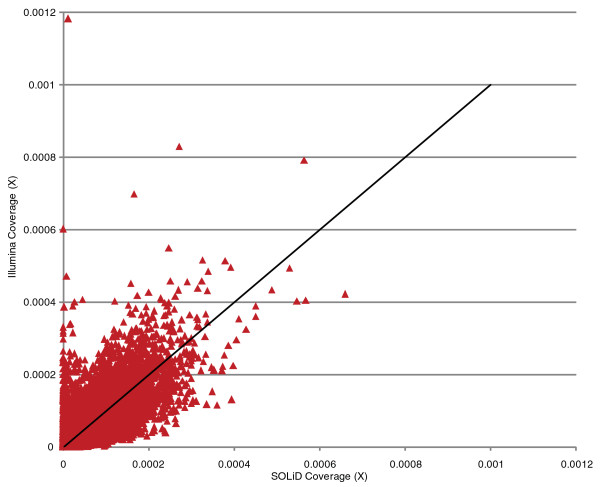
**Average coverage for each target region as sequenced by Illumina 75-bp frag reads plotted against SOLiD library 1**. Coverage for each target is normalized for the total sequence aligned to target. The line X = Y indicates approximately equal levels of coverage in both libraries.

To test the merits of using PE reads versus frag reads we generated both types of library for Illumina sequencing. Target coverage was slightly more uniform for Illumina than SOLiD; however, both sequence types provide more uniform coverage than other solution capture methods (Figure S1 in Additional file 1). Although the majority of targets had similar normalized coverage when sequenced by SOLiD or Illumina, approximately equal numbers showed some coverage-bias depending on sequencing platform (Figure 2). Targets with very different coverage levels typically had low complexity and/or high GC content. PE sequencing showed both a higher percentage of reads on target and significantly fewer duplicate reads (Table 3). In PE sequencing, reads are generated from both ends of a captured DNA fragment and because the approximate fragment length is known, this information can be used to constrain the alignment of both reads to the human genome. Constraining read alignment can greatly improve accuracy when compared to frag sequencing and we hypothesized that these inherent advantages of mapping PE versus single end reads resulted in the increased number of reads derived from the target region. We also suspected that the drastic reduction in the duplicate read rate was not because of a difference in library construction but instead the result of improved informatic identification of 'true' duplicates. Deep, single-end, frag sequencing quickly saturates the target regions such that any additional reads will likely perfectly overlap an existing read and be identified as a duplicate, even when the reads derive from different DNA molecules. PE sequencing, in contrast, allows us to use information about both the start and the end of the capture-DNA fragment in order to determine whether the data are derived from independent DNA molecules. Thus, the increased information content of PE data allows us to reduce the misidentification of duplicate reads.

**Table 3:** Alignment statistics for Illumina PE and frag sequencing libraries

	Illumina Frag	Illumina PE
Total reads aligned	33,524,973	37,832,835
Total data aligned (Gbp)	2.51	2.84
Reads on target (%)	67.62	78.0
Duplicate reads (%)	30.97	8.3
Mean coverage (X)^a^	24	52
Median coverage (X)^a^	20	40
Targets hit (%)	99.36	99.6
Bases ≥1× Coverage (%)	96.39	98.9
Bases ≥10× Coverage (%)^a^	71.33	90.8
Bases ≥20× Coverage (%)^a^	51.23	76.9

^a^Calculated after duplicate read removal.

To test these theories we also analyzed the PE data as if they were generated from a single-ended frag library. This caused the on-target alignment rate to drop slightly to 73% and the duplicate rate to nearly quadruple to 27.6%, virtually identical to the Illumina frag library duplicate rate. The net effect of using PE data instead of frag data was a significant increase in on-target coverage, which resulted in >90% of the targeted bases covered at 10-fold or higher using just 2.8 Gbp of data, a single 2 × 75 bp lane of Illumina sequencing.

To assess the effect of DNA sequencing coverage depth on our ability to correctly identify variants in the exonic region of NA12812, we conducted variant discovery using both approximately 3.3 Gbp and approximately 10 Gbp of SOLiD capture data and 2.8 and 2.5 Gbp of Illumina PE and frag data, respectively. Only a subfraction of these data, non-duplicate sequence reads mapped to target regions, was used for variant discovery, and it is these data, not the total, that ultimately affect variant discovery quality. Discovered variants were compared to known HapMap SNPs in this sample and dbSNP. Here, the concordance to HapMap is used to measure the false negative discovery rate, and the proportion of variants discovered that were also present in dbSNP129 was used to approximate the false positive discovery rate. Others have typically found approximately 90% of CCDS variants to be present in dbSNP for Europeans [3] and significant deviation below 90% may indicate an increased false positive discovery rate.

Table 4 shows the total amount of data produced for each sequencing platform, the final amount of data mapped to targets after duplicate removal and the concordance to dbSNP and HapMap. Three giga-base pairs of SOLiD data allowed discovery of approximately 20 K variants in the 36-Mbp total capture region for the CCDS exome. Six giga-base pairs of additional sequence data facilitated the discovery of approximately 20% more variants. In both cases the proportion of variants present in dbSNP129 was high. Concordance to all known HapMap variants that occur in the target region was 82% and 92% for 3 and 10 Gbp of raw data, respectively. Importantly, this statistic includes known SNPs in regions of little or no coverage where no variant call is possible. When we considered only SNPs that occur in regions with >9× coverage, concordance was approximately 95%.

**Table 4:** Variant discovery and HapMap concordance for different sequencing types and varying amounts of sequence data

	Illumina			SOLiD	
		
	Frag	PE	PE (high stringency)	1	1
Bases produced (Gbp)	2.51	2.84		3.4	9.99
Bases on target after duplicate removal (Gbp)	1.04	2.01		0.59	1.72
Total SNPs	21,239	27,953	26,489	19,790	24,077
dbSNP SNPs	19,525	23,745	23,133	18,016	21,350
dbSNP (%)	91.9	84.95	87.3	91.04	88.67
HapMap variant concordance (%)	83.0	96.0	95.8	81.6	92.9
Variant concordance (>9× coverage) (%)	95.5	98.5	98.2	94.5	97.2

**Figure 3:**
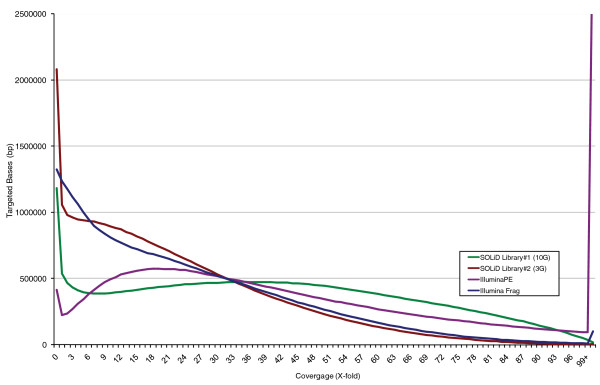
**Coverage distribution across target regions of SOLiD libraries 1 (10 Gbp) and 2 (3 Gbp) and Illumina PE and frag libraries**. The number of bases at each level of coverage for each library type is shown for approximately 10 Gbp of SOLiD data (green), approximately 3 Gbp of SOLiD data (red), approximately 3 Gbp of Illumina PE data (yellow) and approximately 3 Gbp of Illumian frag data (blue) after duplicate removal.

Significantly more variants were discovered in the Illumina PE data than were found in the frag data (Table 4) and consequently there was also higher concordance at HapMap sites. This effect is almost certainly driven by the higher coverage of the target regions achieved by having PE reads. As already noted, this occurs because of a slight increase in reads derived from target, but more significantly, a drastic reduction in the number of reads that are incorrectly marked as duplicates (Figure 3). Interestingly, the proportion of variants that were also in dbSNP was significantly lower in the PE data. This may be due to overall lower read quality at the ends of the PE reads and improved mapping of reads that would not have been aligned without a mate. Increasing the variant calling stringency (LOD = 8; see Materials and methods) on the PE data reduced the number of HapMap concordant variants slightly, but improved the percentage of variants in dbSNP by approximately 3% (Table 4). Although the Illumina PE data also had higher HapMap concordance than the SOLiD variant calls, Illumina frag data performed only slightly better than the SOLiD data, despite having significantly more sequence data on target. When HapMap heterozygous SNP concordance was considered as a function of coverage, SOLiD data out-performed Illumina data at low (<9×) coverage (Figure S2 in Additional file 1); however, Illumina consistently obtained 2 to 3% higher concordance at ≥9× coverage. The quality of the variant calls in both data sets was very high, with 22,066 (91.6%) of variants shared between SOLiD 10 G and the Illumina PE data sets.

This work demonstrates the practicality of genomic target enrichment using capture-sequencing in solution. For the first time, this technology is used at the scale of the whole exome, comprising over 36 Mbp across >170,000 K individual targets. Using four technical replicate libraries, we show that the average coverage of the targeted regions is highly correlated. Capture performance is also shown to be consistent, with the average coverage of each target having >98% correlation between technical replicates. Thus, it is practical to obtain consistent sequence coverage distributions and reproducible variant discovery for a variety of genomic screening experiments. This work also shows the feasibility of using either SOLiD or Illumina-based sequencing after capture. PE data were shown to be superior to frag data, increasing both the on-target number of reads, and greatly improving the correct identification of duplicates. Development of PE sequencing on the SOLiD platform should show a similar effect. Interestingly, Illumina sequencing consistently shows higher levels of enrichment than SOLiD sequencing. This is unexpected because both sequencing platforms yield similar coverage distributions in whole genome sequencing data [12], that is, without enrichment. Further, the capture-sequencing protocols for both methods are almost identical; therefore, we suspect that differences in efficiency are due to an increase in initial library complexity arising from better annealing efficiencies of the Illumina adapter. This is probably explained by the fact that Illumina adaptor sequences contain an A/T overhang, whereas the SOLiD adapters rely on less efficient blunt-end ligation. We are currently developing an A/T overhang-type SOLiD sequencing adapter for use in capture to test whether we can improve levels of enrichment for SOLiD-based capture.

Finally, we examine the amount of sequence data required to fully interrogate the single nucleotide variants of the exome in a HapMap sample using either SOLiD or Illumina sequencing. Using approximately 10 Gbp of SOLiD data, approximately 93% of all HapMap variants are discovered and over 88% of all variants are present in dbSNP. Illumina based sequencing, however, discovers 96% of HapMap variants, with approximately 85% of variants in dbSNP, using only 3 Gbp of sequence data. This result is achieved because our Illumina protocol yields higher overall coverage on the target regions even while producing less raw sequencing data. SOLiD variant calling appears to be more sensitive at <9× coverage, typically obtaining 20% higher concordance. Overall performance between both platforms is similar, however, and the majority of the observed difference is likely due to differences in the variant discovery pipeline software.

## Conclusions

Sequence capture in solution is easier to automate, has higher throughput and is less expensive than microarray-based techniques but has not been extensively used because of performance issues. Here we show the high reproducibility and scalability of our capture method and demonstrate that liquid capture can be used in large-scale experiments to yield reliably high levels of coverage that are consistent at a target-by-target level and are similar to microarray-based techniques. Further, we establish that the entire CCDS exome can be interrogated with just 2.8 Gbp of sequence data, approximately 3% of the required data for whole genome shotgun experiments. At this level of cost and scalability, solution capture-sequencing becomes an attractive technique for rare variant discovery in its own right and as follow-up to genome-wide association studies, especially in studies where much of the heritability of the disease remains unexplained [13] and thus may be due to rare mutations [14]. Capture-sequencing in solution reduces the cost and increases the throughput of rare mutation discovery by focusing on coding regions of the genome and will prove to be a significant addition to the geneticist's tool chest.

## Materials and methods

### Sequence data

All sequence data are available from the Short Read Archive with the following accession numbers [SRA012614 to SRA012615].

### Sequence alignment and variant discovery

SOLiD sequence data were aligned using ABI's corona_lite package (version: 4.0r2.0) with a maximum allowed mismatch of 6; all other parameters were set at default. Pileup-style files were generated with samtools [15] and were filtered to require a variant score of at least 40, or 30 and the variant to be on both strands, and present in at least 15% of all reads. Illumina data were aligned using BWA (v 0.5.3) [16]. The base quality was recalibrated using GATK [17] (downloaded 2 October 2009). Variants were discovered with a minimum LOD of 5 (unless otherwise stated), and were filtered with the following recommended parameters: -X AlleleBalance:low = 0.25, high = 0.75 -X ClusteredSnps.

### Library and capture

The experimental procedures for preparation of pre- and post-capture libraries are described in Additional file 1 and are available on-line for the SOLiD [18] and Illumina platforms [19]. Briefly, 5 μg genomic DNA is sheared, end-repaired and ligated with either Illumina (frag or PE) platform-specific or SOLiD TM platform-specific adaptors. The library is amplified by pre-capture LM-PCR (linker mediated-PCR) and hybridized to NimbleGen SeqCap EZ Exome libraries. After washing, amplification by post-capture LM-PCR and a quantitative PCR-based quality check, the successfully captured DNA is ready for sequencing.

### Probe design

The CCDS (build 36.2) exome capture oligonucleotide pool was designed by targeting 174,984 exons of 16,008 high-confidence protein-coding genes in CCDS. Chromosomal coordinates were obtained from the UCSC genome browser (human build hg18). Target exons were padded to a minimum length of 80 bp, and consolidated to remove redundant overlaps. Coordinates for 528 human miRNA genes were obtained from miRBase (release 10), padded by 25 bp on each end, and likewise consolidated. In sum, the coding and miRNA targets comprised 36 Mb of non-redundant sequence, against which 1.9 million probes were selected on the genomic forward strand, with a median probe length of 75 bp and median start-to-start spacing of 34 bp. A rebalancing algorithm (described in Additional file 1) was used to improve uniformity of coverage across target exons. Probe pools were manufactured by Roche NimbleGen (Madison, WI, USA).

## Abbreviations

bp: base pair; CCDS: Consensus Coding Sequence; frag: fragment; Gbp: giga-base pairs; Mbp: mega-base pairs; miRNA: microRNA; PE: paired end; SNP: single nucleotide polymorphism.

## Authors' contributions

MNB aided in experimental design, sequence alignment and analysis and drafted the manuscript. MW, YQW, IN, DM, and CK conducted the capture hybridization and sequencing. DLB and JDJ participated in experimental design and analysis and aided in manuscript preparation. MJR, MD'A, JK, and TAR participated in designing the capture reagent. TJA and RG participated in experimental design and aided in manuscript preparation.

## Supplementary Material

Additional file 1 **Table S1 and Figures S1 and S2 as well as detailed materials and methods**. Click here for file
