# Supplementary material for: Whole exome capture in solution with 3 Gbp of data
Source: Genome Biol. 2010 Jun 17;11(6):R62. doi: 10.1186/gb-2010-11-6-r62 (PMC2911110; doi:10.1186/gb-2010-11-6-r62)
Supplement: Additional file 1 — Table S1 and Figures S1 and S2 as well as detailed materials and methods. [file gb-2010-11-6-r62-S1.DOC]

Supplementary Information

Supplementary Table 1. Total data produced for each sequence type.

| Library ID | Sequence Type | Total Data Aligned (Gbp) |
| --- | --- | --- |
| 1* | SOLiD 50bp fragment | 9.99 |
| 2* | SOLiD 50bp fragment | 2.67 |
| 3* | SOLiD 50bp fragment | 2.32 |
| 4* | SOLiD 50bp fragment | 2.33 |
| 5 | Illumina 75bp-paired end | 2.87 |
| 6 | Illumina 75bp-fragment | 2.51 |

*Technical replicate libraries

**Supplementary Figure 1.** Cumulative distribution of base coverage shown as a proportion of the mean coverage of all targeted bases. Coverage is more uniform than other solution capture techniques, with approximately 36 and 37% of targeted bases having average coverage or less. This compares favorably to Gnirke et al, wherein only 30% of the bases are covered at the mean level or less, indicating that some target regions show over-coverage at the expense of others.


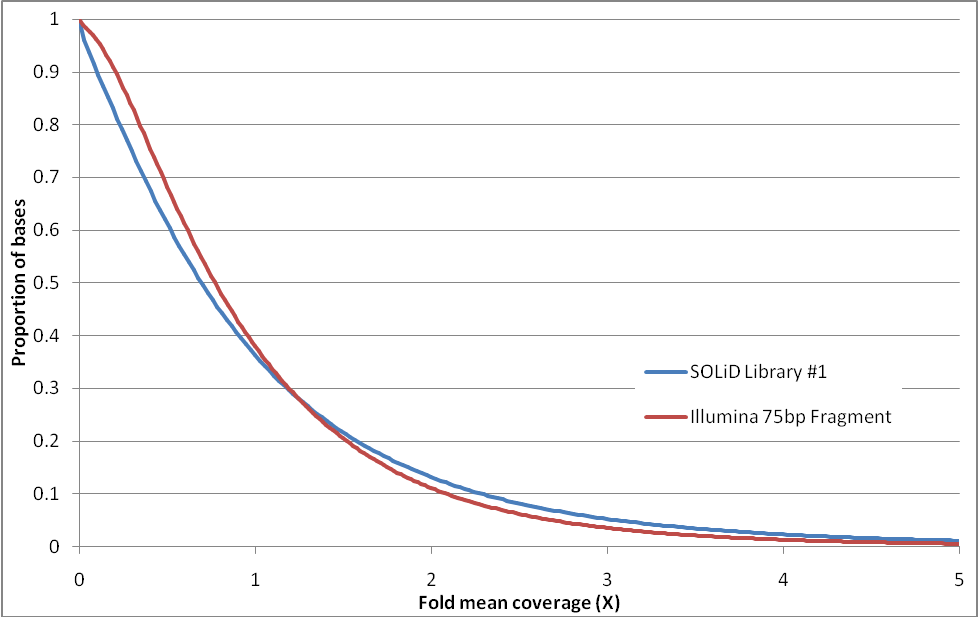


Supplementary Figure 2. SNP discovery concordance to HapMap as a function of coverage from 1x to 60x+ for (a) Solexa-PE reads and (b) SOLiD-fragment reads.

Supplementary Methods

**Pre-Capture Library Preparation**

HapMap sample NA12812 was obtained from the Coriell cell repository. Promega control sample was received from Roche.

For Illumina fragment and paired-end pre-capture library preparation, we follow Illumina Protocol *Preparing Samples for Sequencing Genomic DNA, March 2008* (for fragment library) and *Preparing Samples for Paired-end Sequencing, June 2008* (for paired-end library) with minor modifications. In brief, 5 μg of Hapmap NA12812 DNA or Promega control DNA were nebulized at 45 psi for 3 minute. The nebulized DNA were cleaned up using QIAquick PCR purification kit and later eluted with 30 μl of buffer EB. 1 μl per sample was loaded onto an Agilent Bioanalyzer 2100 DNAChip 7500 for size confirmation. The fragmented DNA samples were then end-polished and 5’-phosphorylated using T4 DNA polymerase, Klenow DNA polymerase and T4 polynucleotide kinase. The end-repaired DNA samples were cleaned up with QIAquick PCR Purification Kit and went through 3’-adenylation using Klenow(exo-) polymerase. After cleaned up with Qiagen MinElute PCR Purification Kit, the 3’-adenylated DNA samples were ligated with Illumina adaptor oligo mix for fragment library preparation or PE adaptor oligo mix for pair-end library preparation. The ligated DNA libraries were purified using QIAquick PCR Purification Kit and were eluted in 30 μl of EB. The eluted ligation products were then loaded onto 2% agarose gels and fragments with size range of 200bp-300bp were cut from the gel and purified using Qiagen Gel Extraction Kit. Elute the DNA in 30 μl of buffer EB. Pre-capture library amplifications by Linker Mediated PCR (LM-PCR) were performed using 5 μl of the eluted DNA and after 12 cycles of amplification, PCR products were cleaned up using QIAquick PCR Purification Kit. The concentration and size distribution of the amplified pre-capture library DNAs were determined by NanoDrop and Agilent Bioanalyzer 2100 DNA Chip 7500.

Based on the section 2.2, *Prepare an express fragment library* in *Applied Biosystems SOLiD™ 3 Plus System Library Preparation Guide (version Oct 2009),* we have generated a modified protocol for SOLiD fragment library preparation for NimbleGen capture and the protocol has been posted in our website http://www.hgsc.bcm.tmc.edu/cascade-tech-solid_capture_protocol-st.hgsc?pageLocation=solid_capture_protocol. In brief, 5 μg genomic DNA is sheared into fragments by Covaris 2 System. After end repair and ligation with SOLiD ^TM^ System platform-specific adaptors, the library DNA was nick-translated and amplified by 10 cycles of pre-capture LM-PCR. PCR products were purified using Agencourt® AMPure® Kit and eluted in 50 μl of buffer EB. The concentration and size distribution of the amplified pre-capture library DNAs were determined by NanoDrop and Agilent Bioanalyzer 2100 DNA Chip 7500.

Liquid phase sequence capture, Washing and Elution

2 μg of the SOLiD pre-capture library DNA or 1 μg of Illumina fragment (or paired-end) pre-capture library DNA were hybridized to the NimbleGen SeqCap EZ Exome Library, followed by washing and elution based on the Roche NimbleGen protocol *NimbleGen SeqCap EZ Exome Library SR User’s Guide (version 1.0)* with minor modifications*.* For SOLiD fragment library, Illumina PE HE1 and PE HE2 Hybridization enhancing oligos in the *User’s Guide* were replaced by SOLiD-A and SOLiD B Hybridization enhancing oligos (refer to our SOLiD protocol for oligo sequence information, posted at http://www.hgsc.bcm.tmc.edu/cascade-tech-solid_capture_protocol-st.hgsc?pageLocation=solid_capture_protocol). For Illumina fragment library, the Illumina PE HE2 oligo was replaced by Hybridization Enhancing oligo with the following sequence: 5’- CAA GCA GAA GAC GGC ATA CGA GCT CTT CCG ATC T-3’.

**Post-capture Linker Mediated PCR (LM-PCR) and Purification**

Captured Hapmap NA12812 and Promega control DNA libraries were amplified by post-capture LM-PCR following our modified SOLiD protocol http://www.hgsc.bcm.tmc.edu/cascade-tech-solid_capture_protocol-st.hgsc?pageLocation=solid_capture_protocol and Roche NimbleGen protocol *NimbleGen SeqCap EZ Exome Library SR User’s Guide (version 1.0).* For SOLiD libraries, we used 12 cycle for post-cap LM-PCR amplification and the amplified post-capture DNA libraries were cleaned up with Qiagen MinElute® PCR purification kit. For Illumina fragment or pair-end libraries, we used 20 cycle for post-cap LM-PCR amplification and the amplified post-capture library DNAs were cleaned up with Qiagen QIAquick PCR Purification kit. After purification, we determined the concentration by Nanodrop. Size distribution of the libraries was confirmed by Agilent Bioanalyzer 2100 DNA Chip 7500.

Quantitative PCR (qPCR)

Quantitative PCR assays using SYBR Green were performed on a standardized set of 4 control loci. Follow Roche NimbleGen protocol *NimbleGen SeqCap EZ Exome Library SR User’s Guide (version 1.0)* for information*.* The Cts were compared for enriched and non-enriched samples and fold enrichment calculated. Samples that successfully enriched (usually ΔΔCt > 7) were put through for sequencing.

Rebalancing Algorithm

The capture oligonucleotide design algorithm comprised two steps: probe design and probe selection. In the first step, a large number of candidate probes were generated genome-wide and screened for suitable hybridization properties such as melting point, G+C content, sub-word frequency, and near-uniqueness (<10 copies), as previously described for microarray capture applications (Albert et al. 2007). In the second step, a certain number of probes in each target region were selected from the database of candidates. To determine the optimal number of probes to allocate to each target, a model was applied to predict coverage along the target intervals as a function of the probe distribution among them. Probe allocation was optimized under a generalized linear model of capture target relative abundance as a function of relative capture probe density. After optimization, constrained optimization was applied to distribute a fixed number of total capture probes to achieve a distribution of probes predicted to provide uniform coverage depth. This constrained optimization problem is solved using a greedy algorithm to assign probes to regions in order to reach the desired distribution of target abundances. The algorithm takes several inputs: the fitted model (from the observed data), the desired final distribution of relative abundances over the targets (to achieve a more uniform distribution), and the minimum and maximum probe densities allowable in any interval. The target read distribution is initially set to zero and is proportionally scaled in a stepwise fashion to reach the final desired distribution. At each step, the probe count required to achieve the target read distribution is computed in each interval subject to the maximum and minimum probe density constraints. The algorithm terminates when the full count of available probes have been allocated.
